# Geoherbalism Metabolomic Analysis of *Atractylodes lancea* (Thunb.) DC. by LC-Triple TOF-MS/MS and GC-MS

**DOI:** 10.3390/molecules28165974

**Published:** 2023-08-09

**Authors:** Hailong Qiu, Chenxiao Shan, Chenghao Fei, Ping Xue, Yongyi Zhou, Jiahuan Yuan, Xin Liu

**Affiliations:** 1 College of Pharmacy, Nanjing University of Chinese Medicine, Nanjing 210023, China; 2 Institute of Chinese Medicinal Materials, Nanjing Agricultural University, Nanning 210095, China; 3 Changzhou Institute for Food, Drug and Fiber Control, Changzhou 213000, China

**Keywords:** *Atractylodes lancea* (Thunb.) DC., geoherbalism, compound separation, GC-MS, LC-triple TOF-MS/MS

## Abstract

The rhizome of *Atractylodes lancea* (Thunb.) DC. (AL), called Maocangzhu in Chinese, is a geoherbalism medical herb in Jiangsu Province that is often used in the prescription of traditional Chinese medicine (TCM), such as for the treatment of COVID-19. The landform and climatic environment of each province varies greatly from south to north, which has an important influence on the chemical constituents in AL. However, there is a lack of research on the significance of its geoherbalism, especially in water-soluble parts other than volatile oil. In this study, eight known compounds were isolated and obtained as reference substances from AL. In addition, liquid chromatography coupled with triple-quadrupole time-of-flight tandem mass spectrometry (LC-triple TOF-MS/MS) and gas chromatography–mass spectrometry (GC-MS) were used to analyze and characterize chemical constituents from different habitats. Moreover, orthogonal partial least-squares discriminant analysis (OPLS-DA) was applied to reveal the differential metabolomics in AL from different habitats based on the qualitative information of the chemical constituents. Results showed that a total of 33 constituents from GC-MS and 106 constituents from LC-triple TOF-MS/MS were identified or inferred, including terpenoids, polyacetylenes, and others; meanwhile, the fragmentation pathways of different types of compounds were preliminarily deduced from the fragmentation behavior of the major constituents. According to the variable importance in projection (VIP) and *p*-values, only one volatile differential metabolite was identified by GC-MS screening: β-eudesmol. Overall, five differential metabolites were identified by LC-triple TOF-MS/MS screening: sucrose, 4(15),11-eudesmadiene; atractylenolide I, 3,5,11-tridecatriene-7,9-diyne-1,2-diacetate, and (3Z,5E,11E)-tridecatriene-7,9-diynyl-1-*O*-(E)-ferulate. This study provides metabolomic information for the establishment of a comprehensive quality evaluation system for AL.

## 1. Introduction

*Atractylodes lancea* (Thunb.) DC. (AL), which is known as Maocangzhu in traditional Chinese medicine, has been reported to be able to remove dampness, invigorate the spleen, and to dispel pathogenic wind and cold effects [1]. The plant is mainly distributed in the provinces of Jiangsu, Hubei, Anhui, and Henan in China. The Maoshan area in Jiangsu is the geoherbalism medicinal herb area of AL. From the external appearance, the cut section of AL has the characteristics of vermilion oil spots and precipitates white frost after being placed for a period of time; however, less of this frosting feature can be observed in the cut section in AL from the Maoshan area. Hence, distinguishing the differences in the chemical constituents of AL is also extremely necessary and important. 

Previously, the focus of research was mainly on the content or ratio of some of the main volatile components. Some reports showed that a specific proportion relationship of its main volatile components existed in AL from the Maoshan area [2], and the content of atractylone was relatively high. However, some others showed that the content of atractylone in AL was low [3]. Moreover, there are also inconsistent reports on the content of atractylodin in the geoherbalism area of AL [4,5], which was used as a quality control indicator in the Chinese Pharmacopoeia. In addition to these main volatile metabolites, AL also contains a large number of other sesquiterpenes, polyacetylenes, and other chemical metabolites. If only the contents of several main chemical metabolites were used as indicators, it would be difficult to reflect the overall effect of traditional Chinese medicine (TCM). The conclusions drawn from the quality evaluation and geoherbalism research of medicinal materials have also been relatively one-sided. However, there has been a lack of in-depth analysis of the differential chemical metabolites among the overall metabolites. Therefore, it is of great significance to clarify the characterization of chemical metabolites of AL for better quality control of medicinal materials. 

In our continuing research, eight known compounds were isolated and obtained as reference substances from AL. These compound structure types were identified as polyacetylene (**1**), triterpene (**2**), sesquiterpenes (**3**–**4**, **8**), steroid (**5**), and diterpenoids (**6**–**7**) by detailed spectroscopic means [6,7,8,9,10,11,12,13]. The two diterpenoids were first isolated from this genus, which updated the phytochemical classification difference between the *Atractylodes* and *Atractylis* genera among Flora Reipublicae Popularis Sinicae [14]. The above compounds were used as standard references for further analyzing secondary metabolites in this plant. 

In recent years, LC-MS and GC-MS techniques have become the most widely used analytical methods for the direct identification of multiple metabolites in TCM [15,16]. Thus, in this experiment, LC-Triple TOF-MS/MS and GC-MS were used to qualitatively analyze AL samples. A total of 106 overall metabolites and 33 volatile metabolites were identified. Based on the above qualitative results, five differential overall metabolites and one volatile metabolite were identified based on VIP and *p*-values. Therefore, our study could explain the geoherbalism of AL from both the overall and volatile aspects, which could be beneficial for the breeding and cultivation of geoherbalism resources of AL in the future. 

## 2. Results

### 2.1. Identification of Isolated Compounds

The known compounds (**1**–**8**) were identified as atractylodin (**1**) [6], taraxerol acetate (**2**) [7], hinesol (**3**) [8], β-eudesmol (**4**) [9], β-sitosterol (**5**) [10], ent-kauran-16β-ol-19-al (**6**) [11], 16α-hydroxy-(-)-kauran-19-oic acid (**7**) [12], and cryptomeridiol (**8**) [13] by NMR analysis and comparison of their spectral data with literature values.

Compound **1:** ^1^H-NMR (500 MHz, CDCl_3_): 7.38 (1H, d, *J* = 1.6 Hz, H-4′), 6.79 (1H, d, *J* = 16.0 Hz, H-1), 6.42 (1H, dd, *J* = 3.4, 1.8 Hz, H-3′), 6.37 (1H, d, *J* = 3.3 Hz, H-2′), 6.36–6.28 (1H, m, H-8), 6.11(1H, d, *J* = 15.9 Hz, H-2), 5.64–5.56 (1H, m, H-7), 1.83 (3H, dd, *J* = 6.9, 1.8 Hz, H-9). ^13^C-NMR (500 MHz, CDCl_3_): 151.96 (C-1′), 143.71 (C-8), 143.58 (C-4′), 130.81 (C-1), 112.16 (C-2′), 111.15 (C-3′), 110.03 (C-7), 104.93 (C-2), 82.00 (C-6), 80.31 (C-3), 77.26 (C-4), 72.64 (C-5), 19.04 (C-9).

Compound **2:** ^1^H-NMR (500 MHz, CDCl_3_): 5.53 (dd, *J* = 8.1, 3.1 Hz, 1H), 4.46 (dd, *J* = 10.5, 5.5 Hz, 1H), 2.04 (s, 3H, H-1′), 2.02 (m, 1H, H7b), 1.92 (dd, 1H, H16b), 1.66 (m, 1H, H11b), 1.64 (m, 1H, H16a), 1.63 (m, 1H, H1b), 1.62 (s, 2H, H12), 1.59 (m, 2H, H2), 1.57 (s, 1H, H6b), 1.49 (s, 1H, H11a), 1.46 (d, *J* = 5.5 Hz, 1H, H6a), 1.42 (m, 1H, H9), 1.38 (d, 1H, H22b), 1.36 (s, 1H, H7a), 1.33 (s, 1H, H21b), 1.31 (d, 1H, H19b), 1.26 (m, 1H, H21a), 1.09 (s, 3H, H26), 1.02 (m, 1H, H22a), 1.00 (m, 1H, H1a), 0.98 (m, 1H, H19a), 0.96 (m, 1H, H18), 0.95 (s, 6H, H25, 29), 0.91 (s, 3H, H30), 0.90 (s, 3H, H27), 0.88 (s, 1H, H5), 0.87 (s, 3H, H23), 0.86 (s, 3H, H24), 0.82 (s, 3H, H28). ^13^C-NMR (500 MHz, CDCl_3_): 15.6 (C-25), 16.69 (C-24), 17.62(C-11), 18.8 (C-6), 21.39 (-CH3), 21.41 (C-27), 23.57 (C-2), 26.03 (C-26), 28.08 (C-23), 28.91 (C-20), 29.94 (C-28), 30.03 (C-30), 33.21 (C-21), 33.45 (C-29), 33.79 (C-12), 35.23 (C-22), 35.89 (C-17), 36.77 (C-19), 37.5 (C-13), 37.67 (C-1), 37.79 (C-4), 37.8 (C-16), 38 (C-10), 39.1 (C-8), 41.34 (C-7), 48.88 (C-18), 49.31 (C-9), 55.75 (C-5), 81.13 (C-3), 117.04 (C-15), 158.08 (C-14), 171.08 (-CO).

Compound **3:** ^1^H-NMR(500 MHz, CDCl_3_): 0.92 (d, *J* = 6.8 Hz, 3H, C10-CH_3_), 1.20 (s, 6H, C_2_-2CH_3_), 1.27–1.78 (m, 10H, -CH-), 1.68 (s, 3H, C6-CH_3_), 1.90–1.97 (m, 3H, -CH-), 5.31 (m, 1H, C7-H). ^13^C-NMR (500 MHz, CDCl_3_): 140.13 (C-10), 121.76 (C-1), 72.07 (C-11), 51.49 (C-7), 49.56 (C-5), 36.79 (C-4), 35.78 (C-9), 33.34 (C-6), 28.49 (C-13), 28.03 (C-8), 27.77 (C-12), 27.29 (C-3), 24.28 (C-2), 19.98 (C-15), 16.27 (C-14).

Compound **4:** ^1^H-NMR (500 MHz, CDCl_3_): 0.7 (s, 3H, H-15), 1.20 (s, 6H, H-13, H-14), 1.77 (d, 1H, H-5), 1.98 (m, 1H, H-3a), 2.31 (d, 1H, H-3b), 4.44 (d, 1H, H-11a), 4.71 (d, 1H, H-11b). ^13^C-NMR (500 MHz, CDCl_3_): 41.21 (C-1), 23.58 (C-2), 36.99 (C-3), 151.23 (C-4), 49.53 (C-5), 25.11 (C-6), 49.88 (C-7), 22.47 (C-8), 41.93 (C-9), 35.99 (C-10), 73.01 (C-11), 27.22 (C-12), 27.27 (C-13), 105.42 (C-14), 16.40 (C-15).

Compound **5:** ^1^H-NMR (500 MHz, CDCl_3_): 5.35 (m, 1H), 3.52 (m, 1H), 1.00 (s, 3H), 0.92 (m, 3H), 0.85 (m, 3H), 0.82 (m, 3H), 0.79 (m, 3H), 0.68 (m, 3H). ^13^C-NMR (500 MHz, CDCl_3_): 140.84 (C-5), 121.81 (C-6), 71.89 (C-3), 11.95 (C-18), 19.12 (C-19), 18.87 (C-21), 19.49 (C-26), 19.92 (C-27), 12.07 (C-29), 36.24 (C-1), 29.21 (C-2), 39.85 (C-4), 31.74 (C-7, C-8), 50.20 (C-9), 36.59 (C-10), 19.50 (C-11), 37.34 (C-12), 42.39 (C-13), 56.85 (C-14), 23.14 (C-15), 26.13 (C-16), 56.13 (C-17), 34.02 (C-20), 31.98 (C-22), 24.39 (C-23), 45.90 (C-24), 28.34 (C-25), 21.17 (C-28).

Compound **6:** ^1^H-NMR (500 MHz, CDCl_3_): 0.80 (1H, ddd, H-1a), 0.89 (3H, s, H-20), 0.98 (1H, m, H-9), 1.01 (3H, s, H-18), 1.03 (1H, m, H-3a), 1.17 (1H, m, H-5), 1.40 (3H, s, H-17), 1.46 (1H, d, *J* = 3.8 Hz, H-7a), 1.50 (1H, m, H-12a), 1.52 (1H, m, H-12b), 1.55 (2H, m, H-11), 1.59 (2H, m, H-15), 1.61 (1H, m, H-14a), 1.65 (1H, m, H-2a), 1.68 (1H, m, H-2b), 1.70 (1H, m, H-6a), 1.74 (1H, m, H-7b), 1.82 (1H, m, H-1b), 1.86 (1H, m, H-13), 1.89 (1H, m, H6b), 1.92 (1H, m, H-14b), 2.15 (1H, m, H-3b), 9.76 (1H, d, H-19). ^13^C-NMR (500 MHz, CDCl_3_): 39.83 (C-1), 18.18 (C-2), 34.29 (C-3), 48.56 (C-4), 56.67 (C-5), 20.13 (C-6), 42.04 (C-7), 45.24 (C-8), 55.47 (C-9), 39.47 (C-10), 18.43 (C-11), 26.74 (C-12), 48.95 (C-13), 37.87 (C-14), 57.74 (C-15), 79.41 (C-16), 24.59 (C-17), 24.33 (C-18), 206.03 (C-19) and 16.47 (C-20).

Compound **7**: ^1^H-NMR (500 MHz, CDCl_3_): 0.87 (3H, s), 1.28 (3H, s), 1.58 (3H, s), 2.05 (1H, s). ^13^C-NMR (500 MHz, CDCl_3_): 42.15 (C-1), 20.44 (C-2), 39.42 (C-3), 43.8 (C-4), 57.63 (C-5), 23.46 (C-6), 43.42 (C-7), 46.59 (C-8), 58.31 (C-9), 40.99 (C-10), 19.49 (C-11), 28.00 (C-12), 49.66 (C-13), 38.63 (C-14), 58.63 (C-15), 80.03 (C-16), 24.58 (C-17), 29.64 (C-18), 181.83 (C-19), 16.43 (C-20).

Compound **8:** ^1^H-NMR (500 MHz, CDCl_3_): 1.91 (1H, dd, H-6a), 1.79 (1H, dd, H-3a), 1.60 (1H, m, H-8a), 1.51 (1H, m, H-9a), 1.44 (1H, m, H-1a), 1.36 (1H, m, H-7), 1.33 (1H, m, H-3b), 1.28 (1H, m, H-8b), 1.21 (3H, s, H-12), 1.21 (3H, s, H-13), 1.19 (1H, m, H-5), 1.14 (1H, m, H-9b), 1.11 (3H, s, H-14), 1.06 (1H, m, H-1b), 1.00 (1H, m, H-6b), 0.85 (3H, s, H-15). ^13^C-NMR (500 MHz, CDCl_3_): 41.07 (C-1), 20.23 (C-2), 43.50 (C-3), 72.43 (C-4), 54.84 (C-5), 21.55 (C-6), 49.96 (C7), 22.70 (C-8), 44.65 (C-9), 34.58 (C-10), 73.06 (C-11), 27.41 (C-12), 27.11 (C-13), 18.76 (C-14), 22.59 (C-15).

### 2.2. Analysis of Metabolites by GC-MS

#### 2.2.1. Identification of Metabolites by GC-MS

**Figure 1:**
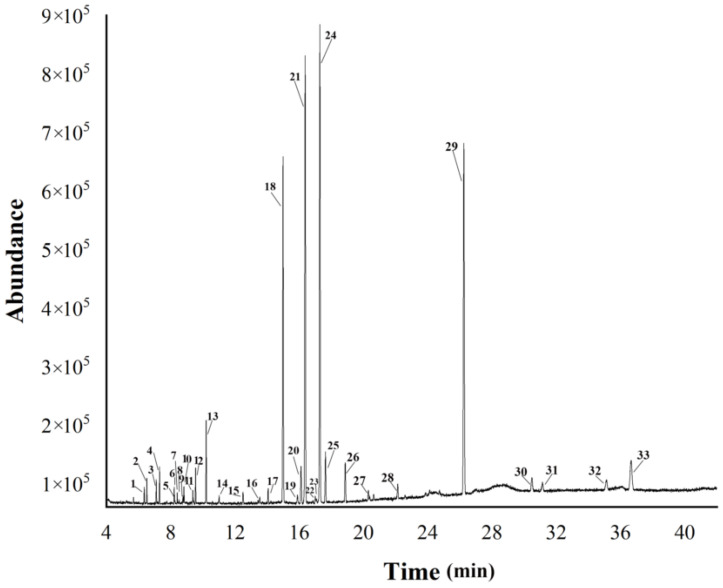
The TIC of AL from the Maoshan area by GC-MS.

A total of 33 variables from matrixes containing retention time, mass-to-charge ratio (*m*/*z*), and peak intensity were detected in the total ion current in a 42 min measurement period. The metabolites were identified as listed in Table 1 and include monoterpene, sesquiterpenes, polyacetylenes, and other compounds. The other signals were very weak or not even included in the database. A characteristic GC-MS total ion current chromatogram of AL from the Maoshan area is shown in Figure 1.

#### 2.2.2. OPLS-DA

**Figure 2:**
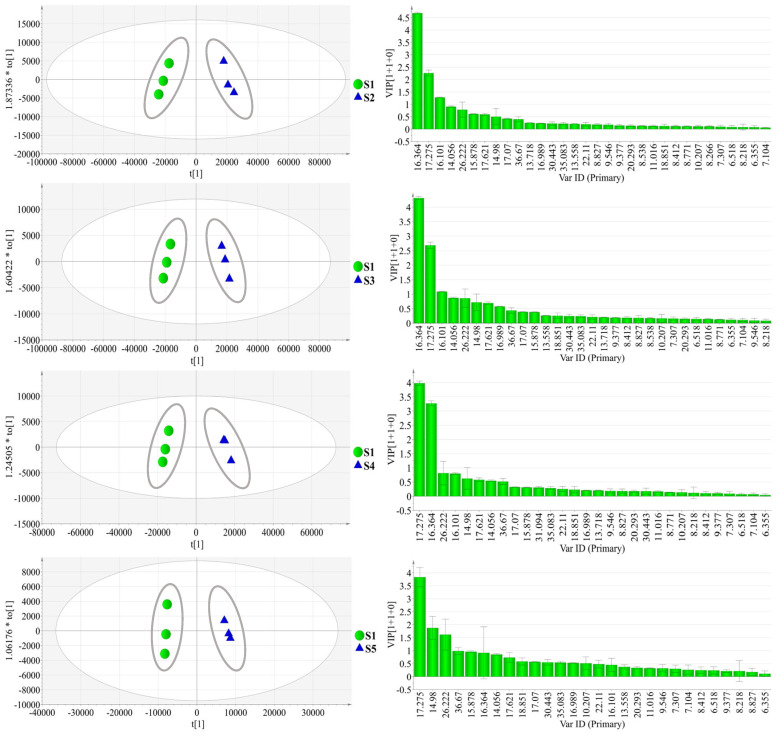

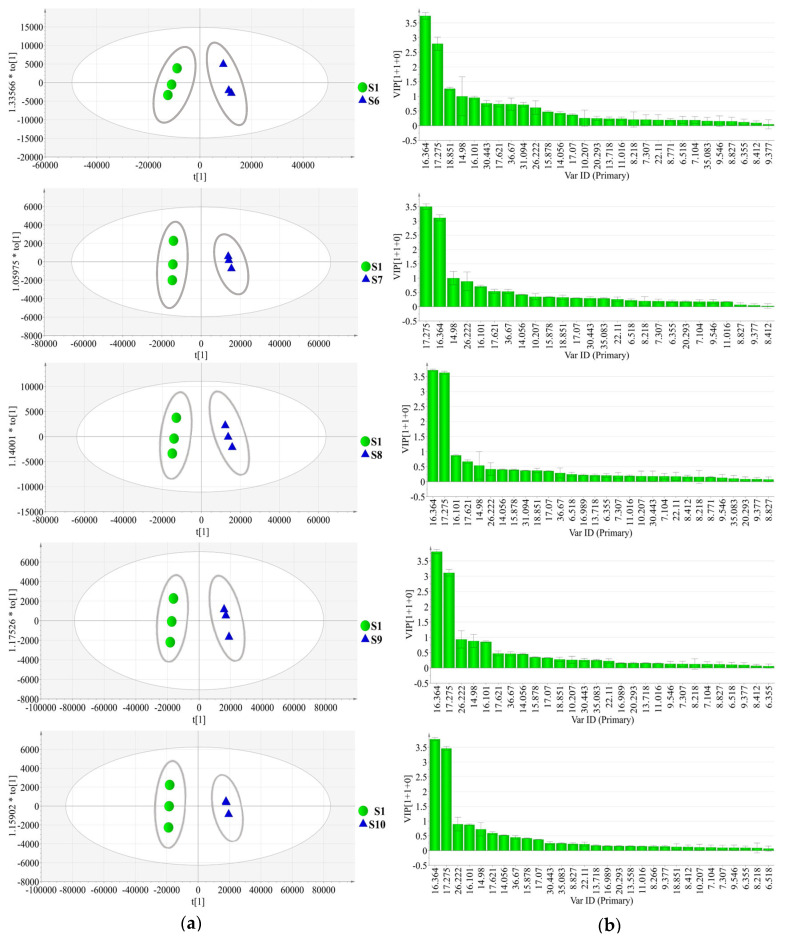
OPLS-DA scatter plot (**a**) and VIP score plot (**b**) of AL samples from different habitats by GC-MS.

OPLS-DA, a discriminant multidimensional statistical analysis method, was consequently utilized to locate the radically differential metabolites among the AL samples in the GC-MS database. This model separated S1 and each of the other samples along the discriminating t [1] (Figure 2). The value of *R*^2^*Y* and *Q*^2^*Y* indicated that this OPLS-DA model could explain the differences between sample groups. Then, the results of permutation tests conducted 200 times showed the value of the *Y*-intercept of *R*^2^ and the *Y*-intercept of *Q*^2^ (Table 2). According to these results, the models are valid and reliable. The VIP scatter plot of OPLS-DA identified the underlying biomarkers of each part, where *p* < 0.05 indicated significant differences. Differential chemical constituents (VIP > 1) of AL samples from different habitats were selected from the screening and the number of characteristic peaks are shown in Table 2.

#### 2.2.3. Identification of the Differential Metabolites

**Figure 3:**
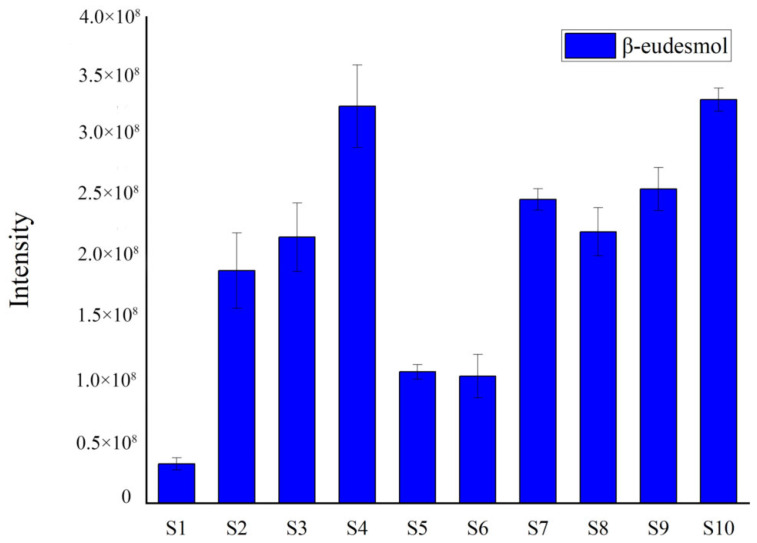
Relative content of the differential chemical constituents by GC-MS.

The results indicated that β-eudesmol was the most contributive principle distinguishing the S1 sample from the other samples of AL. The average value and standard deviation of the peak area of the differential metabolite was calculated to obtain the relative content changes among the different samples (Figure 3). As shown in the figure, the content of β-eudesmol of AL from S1 sample was low.

### 2.3. Analysis of Metabolites by LC-Triple TOF-MS/MS

#### 2.3.1. Identification of the Metabolites in AL

**Figure 4:**
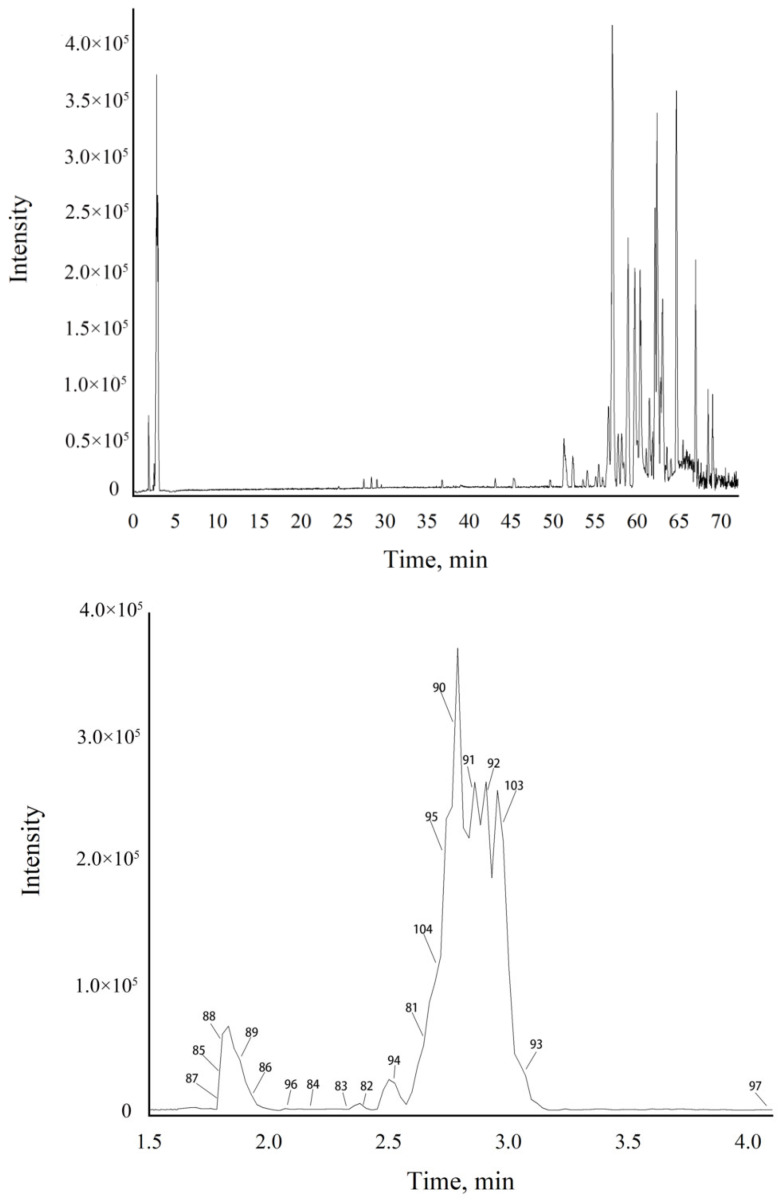

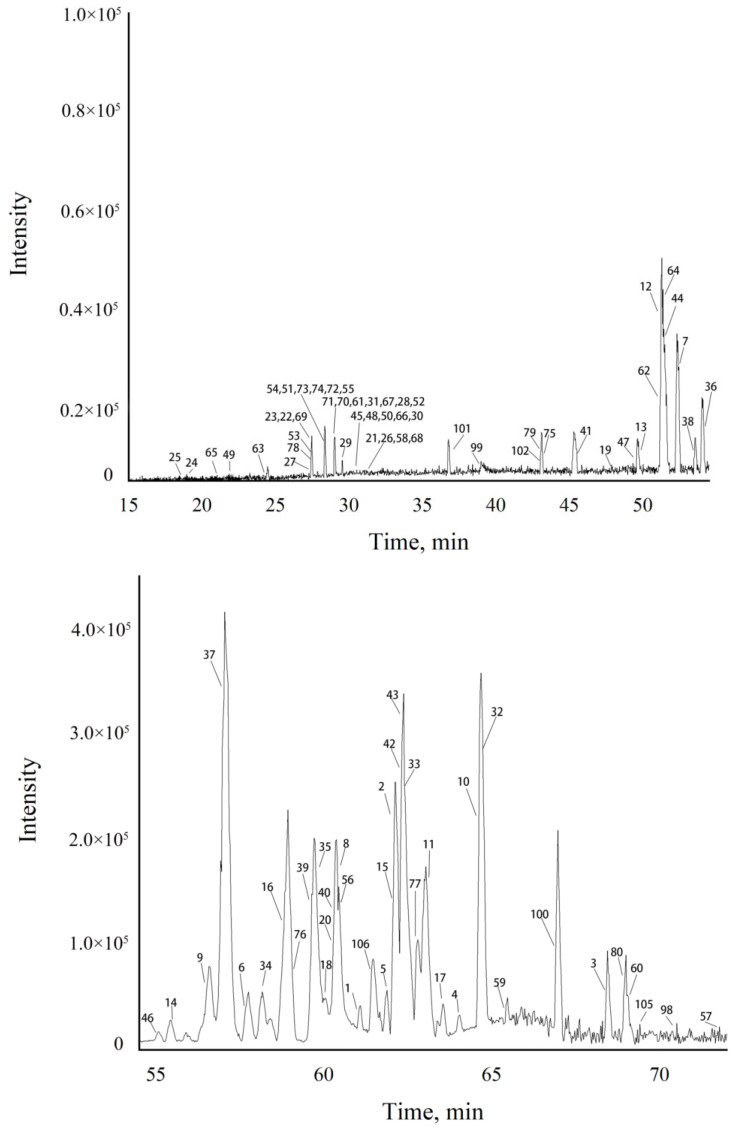
The BPC of AL from QC by LC-Triple TOF-MS/MS in positive-ion mode.

The base-peak chromatogram (BPC) of the QC sample in the positive-ion mode is shown in Figure 4. A total of 106 metabolites were identified, including 32 sesquiterpenes, 22 polyacetylenes, and 52 other metabolites. Due to the fact that more volatile aglycone metabolites existed in AL and that the content of water-soluble glycosides was low, the response values of glycosides were relatively low in the figure. Detailed information on the identified metabolites are shown in Table 3 and Table 4.

##### Identification of Sesquiterpenes

**Figure 5:**
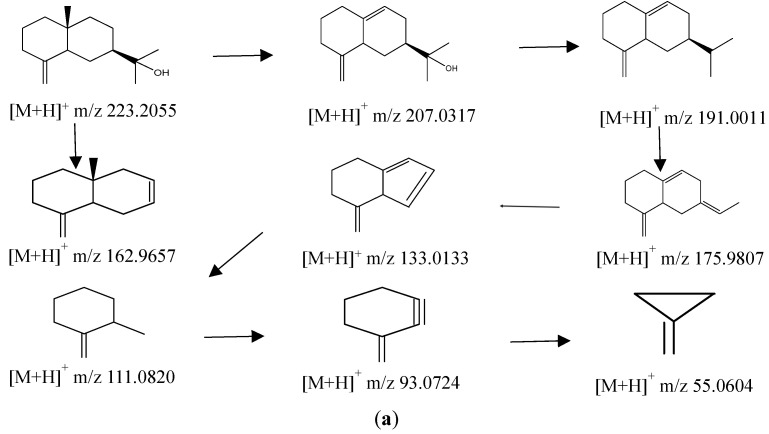

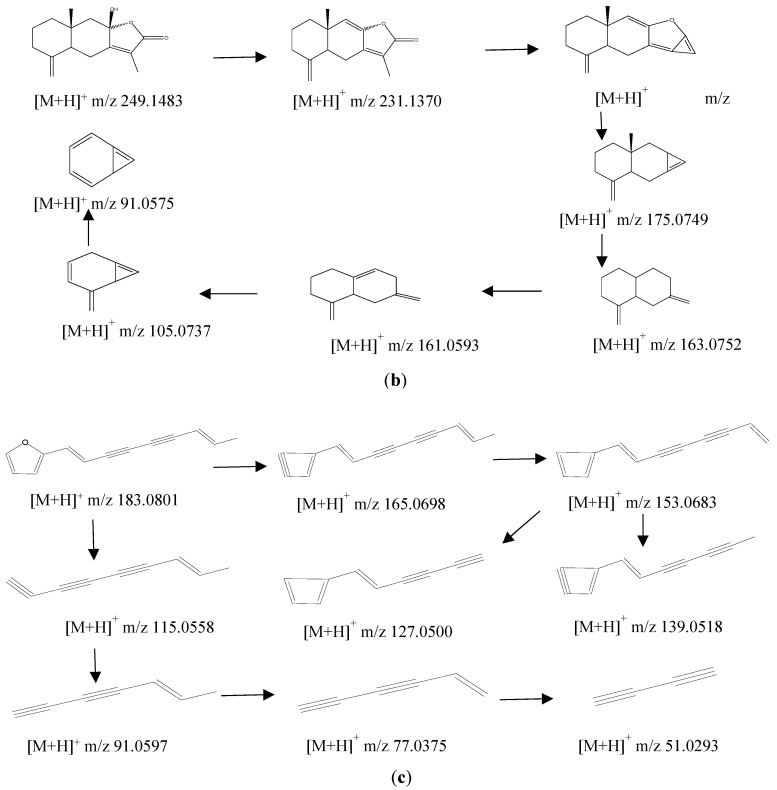
The possible fragmentation pathways of β-eudesmol (**a**), atractylodes III (**b**), and atractylodin (**c**) in AL.

Sesquiterpenes are the main active ingredients of AL. A total of 32 sesquiterpenes were identified in this study, including sesquiterpene aglycone and sesquiterpene glycoside (Table 3). Fragmentation patterns of sesquiterpenes are shown in Figure 5a,b.

Sesquiterpene aglycone: According to the above standard cracking rule, it was concluded that sesquiterpene aglycone preferentially lost H_2_O (18 Da), CH_2_ (14 Da), CH_4_ (16 Da), and C_3_H_8_O side-chain groups (60 Da). In addition, it could be seen from the fragment ions of sesquiterpene lactones that lactones generally lost neutral fragments such as CO (28 Da), CO_2_ (44 Da), and HCOOH (46 Da). In the MS^1^ spectrum, the molecular formula composition of the screened target ions was predicted by using the Formula Finder function of the PeakView software (version 1.2). Once it was predicted to have a C_15_ molecular formula, it would be initially locked in as a potential sesquiterpene aglycone compound. Furthermore, if there were methoxy group and acetic acid substitutions in the structure, the loss of CH_3_OH (32Da) and CH_3_COOH (60 Da) would exist in the MS^2^ of each compound. Taking compound **19** as an example, the quasi-molecular ion peak of 321.1700 [M + H]^+^ was first formed. Fragment ions at *m*/*z* 289.1408, 229.1240, 187.0723, and 159.1154 represented the loss of CH_3_OH (32 Da), CH_3_COOH (60 Da), 3CH_2_ (42 Da), and CO (28 Da). In addition, the left or right ring might be lost in some compounds such as **9** and **18**. Finally, compounds **1**–**19** were identified as sesquiterpene aglycones.

Sesquiterpene glycoside: Compounds **20**–**32** were identified as sesquiterpene glycosides. These sesquiterpene glycosides exhibited some of the same cracking laws in MS^2^, such as the loss of apiofuranosyl (150 Da), xylopyranoside (150 Da), and glucopyranoside (180 Da), while the lost glycosyl fragments might undergo protonation as well. After the loss of glycosyl fragments, the other parts were mainly sesquiterpene aglycones, which followed a similar cracking rule as above. In the MS^1^ spectrum, the molecular formula composition of the screened target ions was predicted by using the Formula Finder function of the PeakView software (version 1.2). Once it was predicted to have the C_20_ (linked pentose), C_21_ (linked hexose), C_25_ (linked two pentose, less), C_26_ (linked one pentose and one hexose), and C_27_ (linked two hexose) molecular formula, it would be initially locked in as a potential sesquiterpene glycoside compound. According to the above fragmentation rules and literature reports, compounds **20**–**32** were identified as sesquiterpene glycosides.

##### Identification of Polyacetylenes

Polyacetylenes are also the main active ingredients of AL. A total of 22 polyacetylenes were identified in this study, including polyacetylene aglycones, and polyacetylene glycosides (Table 3). Fragmentation patterns of polyacetylene are shown in Figure 5c.

Polyacetylene aglycone: According to the above standard cracking rule, it was concluded that polyacetylene aglycone preferentially lost H_2_O (18 Da), CH_2_ (14 Da), furan ring (*m*/*z* 68), and C_2_H_2_ (*m*/*z* 26). Due to the basic C=C and C≡C structures of polyacetylenes, unsaturated C-chain groups were often lost during fragmentation, which helped to distinguish other types of compounds. According to existing literature reports, acetic acid products mostly exist in polyacetylenes. The MS^2^ spectrum mainly showed the loss of CH_3_COOH (*m*/*z* 60) and CH_3_COONa (*m*/*z* 82), resulting in [M + Na-CH_3_COOH]^+^ or [M + H-CH_3_COONa]^+^ fragment ions. In addition, *m*/*z* 199, 167, and 141 were also reported to be characteristic ions in the cracking process of alkyne components [39]. The above fragmentation patterns and fragment ions contributed to the comprehensive analysis of such compounds. For example, compound **34** formed a mass-to-charge ratio of 199.0760 [M + H]^+^ with an excimer ionization peak (C_13_H_10_O_2_) near the retention time of atractylodin in the MS^1^ spectrum. The MS^2^ fragmentation included *m*/*z* 183, 165, 153, and 115. Referring to the cleavage law of atractylodin, compound **34** had an additional excimer ion peak at 199, which suggested an additional oxygen (16 Da) different from that of atractylodin with *m*/*z* 183. Based on the relevant information reported in the plant literature, compound **34** was preliminarily identified as atractylodinol. Specifically, characteristic fragment ions of *m*/*z* 141 existed in compounds **37**–**41**, a furan ring existed in compounds **34**, **36**, **38**, and **40**, and CH_3_COOH (*m*/*z* 60 or 82) existed in compound **35**–**42**. In addition, ferulic existed in compound **43**.

Polyacetylene glycoside: Compounds **44**–**54** were identified as polyacetylene glycosides. These polyacetylene glycosides exhibited some of the same cracking laws in MS^2^, such as the loss of rhamnose (146 Da), apiofuranosyl (150 Da), and glucopyranoside (180 Da), while the lost glycosyl fragments might undergo protonation as well. After the loss of glycosyl fragments, the other parts were mainly polyacetylene aglycones, which followed a similar cracking rule as above. Taking compound **52** as an example, the quasi-molecular ion peak at 535.1783 [M + Na]^+^ was first formed. Fragment ions at *m*/*z* 335.0952, and 203.0588 represented the ions of [glucopyranoside-apiofuranosyl + Na]^+^ (335 Da) and [glucopyranoside + Na]^+^ (203 Da).

##### Identification of Other Compounds

Compounds **55**–**106** were identified by comparison with standard samples, the mother ions of self-built molecules, the reported literature and the MS^2^ fragment ions (matching ratio of nearly 100%), as well as by the MS-dial software (version 4.9.2) (URL: http://prime.psc.riken.jp/, accessed on 22 October 2022) in combination with its open-source database (URL: http://prime.psc.riken.jp/comp-ms/msdial/main.html#MSP, accessed on 22 October 2022, score > 90). The results are shown in Table 4.

#### 2.3.2. OPLS-DA

**Figure 6:**
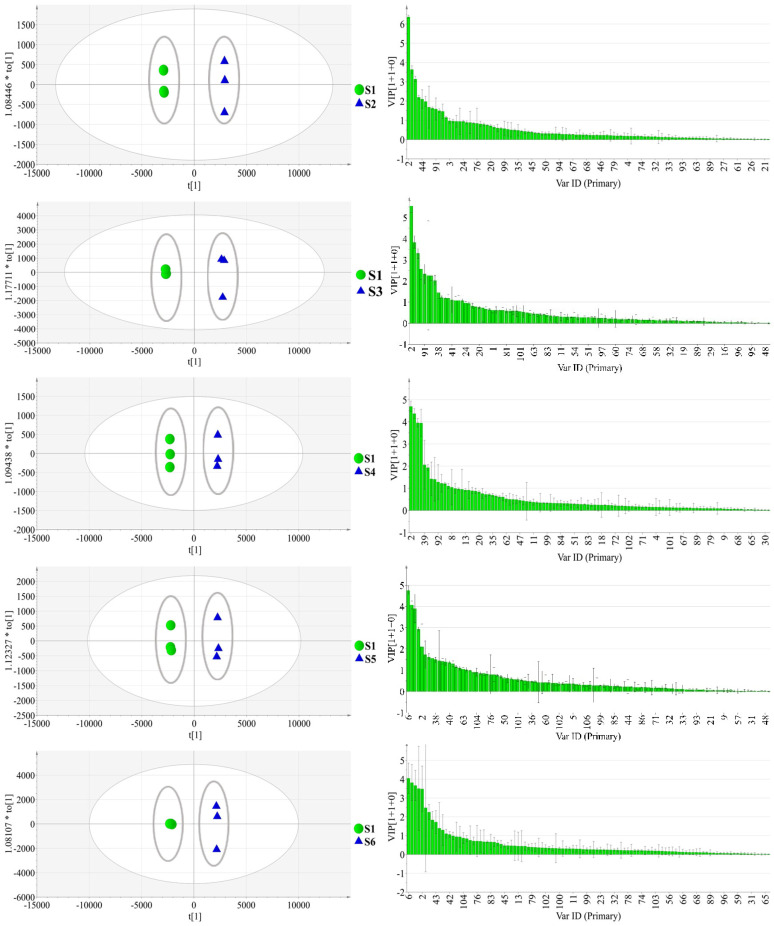

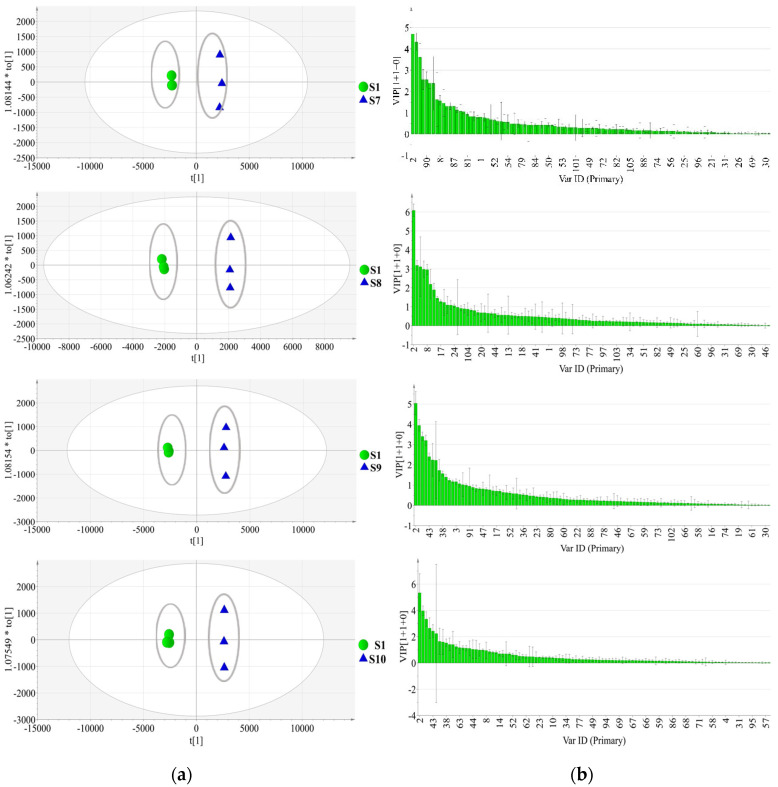
OPLS-DA scatter plot (**a**) and VIP score plot (**b**) of AL samples from different habitats by LC-Triple TOF-MS/MS.

In this experiment, the samples from other habitats were compared with the sample from the Maoshan area and analyzed by OPLS-DA analysis. As can be seen from Figure 6a, the samples from each group were obviously separated along the PC1 axis. The models were tested with 200 permutations, and the results are listed in Table 5. The results showed that the models did not overfit, indicating that they were effective and reliable. According to the VIP score chart (Figure 6b) and *t*-tests corresponding to the model, differential chemical constituents (VIP > 1) were selected out by screening, and the numbers of characteristic peaks are shown in Table 5.

#### 2.3.3. Identification of the Differential Metabolites

A total of five common differential chemical constituents were identified from S1 and other samples of AL: sucrose, 4(15),11-eudesmadiene, atractylenolide I, 3,5,11-tridecatriene-7,9-diyne-1,2-diacetate, and (3Z,5E,11E)-tridecatriene-7,9-diynyl-1-*O*-(E)-ferulate.

**Figure 7:**
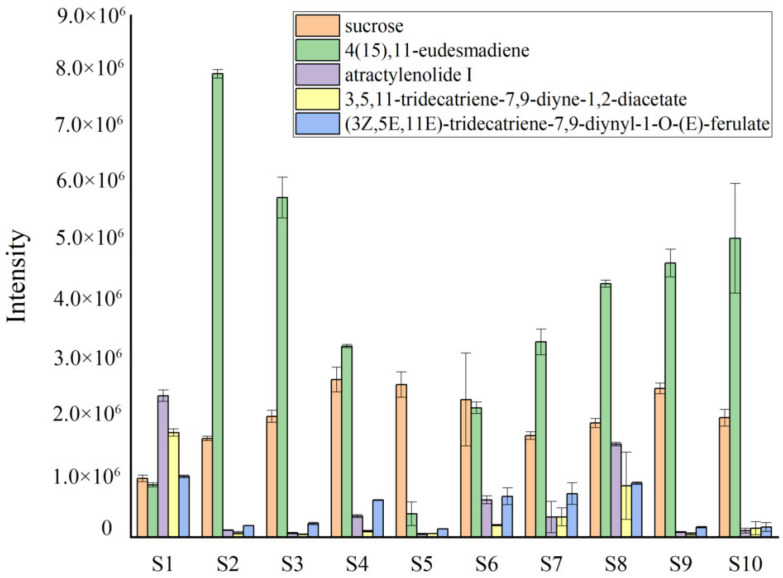
Relative content of the different metabolites by LC-Triple TOF-MS/MS.

The average values of common differential constituents between the different samples were calculated to obtain the content changes (Figure 7). As shown in the figure, the contents of sucrose and 4(15),11-eudesmadiene were lower in S1 than in the other samples, while the contents of atractylenolide I, 3,5,11-tridecatriene-7,9-diyne-1,2-diacetate, and (3Z,5E,11E)-tridecatriene-7,9-diynyl-1-*O*-(E)-ferulate were higher in S1 than the others.

## 3. Discussion

In the past, the focus of geoherbalism research on AL was mainly on the content or ratio of some of the main volatile components; however, the conclusions were inconsistent. The problem mainly focuses on whether the content of atractylone in the Maoshan area is truly higher than that in other habitats. Moreover, in the current Chinese Pharmacopoeia, the quality control indicator of AL is the content of atractylodin, which is unable to fully distinguish whether it is from a geoherbalism area. Not only that, evaluating the quality of Chinese medicinal herbs with only a few main components is not appropriate for determining the overall quality of the TCM. At present, there are few studies on the geoherbalism research on AL, especially for water-soluble metabolites. Meanwhile, the natural resources in the Maoshan area are seriously scarce, suggesting that the breeding and cultivation of excellent geoherbalism varieties are urgent. Therefore, it is of great importance to study the geoherbalism differential metabolites of AL.

In this study, LC-triple TOF-MS/MS and GC-MS methods were used to comprehensively analyze the different metabolites of AL. Using these methods, 33 volatile chemical metabolites (GC-MS) and 106 overall chemical metabolites (LC-triple TOF-MS/MS) in AL were identified, which included sesquiterpenes, polyacetylenes, and others. The results showed that some metabolites of AL in the Maoshan area were significantly different from those in other areas, suggesting that they might be related to the geoherbalism and highly varied quality of AL. In addition, we also found that several overall differential metabolites with higher content in the Maoshan area were also higher in the Yingshan and Tongbai areas (Figure 7), which are also famous AL production areas, indirectly proving the advantage of metabolomics in comprehensively evaluating the quality of medicinal materials from different habitats.

Although the volatile components have been studied and reported, we have provided a new method that can effectively separate multiple components in the volatile oil, especially hinesol and β-eudesmol. The method is also suitable for evaluating other varieties and counterfeits of *Atractylodis* Rhizome, which has certain innovative significance for comprehensively evaluating the quality of Cangzhu. Not only that, we found that the content of atractylone in the Maoshan area was indeed higher than that in other habitats. However, it was not a differential metabolite, indicating that other metabolites may be more meaningful for distinguishing the geoherbalism of AL, which indirectly indicated the necessity of using metabolomics in this experiment.

The Chinese Pharmacopoeia shows that AL has certain dampness-eliminating and spleen-strengthening effects. According to literature reports, its dampness-eliminating effect is mainly related to the content of the volatile oil [40]. AL from the Maoshan area has also been considered to have a stronger spleen-strengthening effect than that from other production areas [41]. In this study, β-eudesmol played a strong contributory role in volatile oil, indicating a strong correlation with the dampness-eliminating effects of AL. However, in terms of overall detection, metabolites such as atractylenolide I contributed significantly, indicating a significant correlation with the spleen-strengthening effects of AL. This also proves the advantages of different metabolomic methods for evaluating the geoherbalism efficacy of AL.

Generally speaking, the accumulation of active ingredients in AL varies greatly according to different ecological environments. Therefore, it is necessary to analyze the relationship between different ecological environments and metabolites. The results provide data that reveal the influence of the ecological environment on the synthesis and accumulation of metabolites in AL as well as the quality formation mechanism of geoherbalism characteristics.

## 4. Materials and Methods

### 4.1. General Experimental Procedures

NMR spectra were recorded on a Bruker DRX-500 spectrometer (Bruker, Karlsruhe, Germany), using CDCl_3_ as solvents. ESI-MS data were obtained on an AB Sciex Triple TOF^TM^ 5600 system-MS/MS (AB SCIEX, Framingham, MA, USA) mass spectrometer. EI-MS data were obtained on an Agilent 7890A/5975C GC-MS (Agilent, J&W Scientific, Folsom, CA, USA) mass spectrometer. Column chromatography was performed on silica gel (100–200 mesh and 200–300 mesh, Qingdao Marine Chemical Factory, Qingdao, China). TLC was performed on precoated silica gel plates (G and GF254, Qingdao Marine Chemical Factory).

### 4.2. Plant Materials

The air-dried rhizomes of AL using for separation and extraction were collected from Huoshan of the Anhui province, China, and the air-dried rhizomes of AL used for metabolomic analyses were collected from different habitats of AL, which were identified as *Atractylodes lancea* (Thunb.) DC. by Prof. Gu Wei. Voucher specimens were deposited in the Laboratory of Chinese Medicine Identification, Nanjing University of Chinese Medicine. The source information of the AL samples is shown in Table 6. All sample batches were randomly sampled into three mixed-batch groups again in order to reduce intra-group errors. All of them were then crushed and immediately extracted.

### 4.3. GC-MS Analysis of AR

#### 4.3.1. Sample Preparation

The experimental procedure for the extract preparation for GC-MS is as follows: Samples were accurately weighed (0.5 g for each sample) and transferred to 50 mL triangular flasks; then, 10 mL cyclohexane was added into each of them, followed by ultrasonic extraction for 20 min. After extraction, the samples were cooled down, weighed, and the weight of each sample was replenished. Finally, the supernatant was taken and centrifuged at 13,000 rpm for 10 min prior to GC-MS analysis.

#### 4.3.2. GC-MS Methods

The Agilent 7890A/5975C GC-MS instrument (Agilent, J&W Scientific, Folsom, CA, USA) was used for analysis. Gas chromatographic conditions were as follows: Agilent 19091N-133 column (30 m × 250 µm, 0.25 µm; Agilent J&W Scientific). Split sampling was performed with an injection volume of 2 μL and a split ratio of 40:1. The injection, ion source, and interface temperatures were set at 280 °C, 250 °C, and 150 °C, respectively. The oven temperature-raising procedure was set to 100 °C for 3 min and then increased by 10 °C/min to 160 °C. After this, the temperature was increased by 3 °C/min to 175 °C and held for 2 min, followed by an increase of 5 °C/min to 200 °C, which was then held for 1 min, and finally, the temperature was increased by 2 °C/min to 210 °C and held for 15 min. The total run time was set for a 42 min measurement period. The carrier gas was helium (1.0 mL/min). Mass spectrometry conditions: electrospray ionization source, electron energy 70 eV; mass data collected in full-scan mode (*m*/*z* 35–780).

#### 4.3.3. Data Preprocessing and Analysis

The raw data of the GC-MS analysis from the Agilent MSD ChemStation was automatically detected and aligned using the NIST database. On the basis of the above qualitative results, OPLS-DA was used to perform dimensionality reduction analysis on the data to obtain information about differences between groups. The characteristic peaks of the differential chemical components were screened according to the VIP (VIP > 1) and *t*-test (*p* < 0.05) results obtained from the OPLS-DA.

### 4.4. LC-Triple TOF-MS/MS Analysis of AL

#### 4.4.1. Preparation of Reference Substances and Sample Solutions

Four reference substances, including atractylenolide I~III and atractylone, were purchased from Shanghai Yuanye Biotechnology Co., Ltd. (Shanghai, China). The other eight reference substances were self-made in this experiment. The above 12 standards were weighed in a 5 mL volumetric flask using a 1/1,000,000 electronic analytical balance (ME36S, Sartorius, Göttingen, Germany), dissolved in methanol, and prepared into a mixed standard solution. All solutions were stored at 4 °C for further analysis.

The samples were crushed into 40 meshes using a universal grinder, and then the powder was dried to constant weight. Next, 0.5 g of dried powder was accurately weighed into a 50 mL triangular flask and ultrasonically extracted with 10 mL of methanol for 60 min at room temperature. After extraction for a few minutes, the weight was made up with methanol. The supernatant was taken and centrifuged at 13,000 rpm for 10 min before filtering through a 0.22 µm membrane (Jinteng Laboratory Equipment Co., Ltd., Tianjin, China) prior to LC-triple TOF-MS/MS analysis.

#### 4.4.2. LC-Triple TOF-MS/MS Conditions

An LC system (Shimadzu, Kyoto, Japan) was used for sample analysis. The separation was conducted by an ODS C18 analytical column (4.6 mm × 250 mm, 5 µm; GL sciences, Inc., Tokyo, Japan) at 30 °C. The mobile phase consisted of 0.1% formic acid in water (A) and methanol (B) with the following gradient elution: 0–5 min (10–14% B), 6–22 min (14–30% B), 22–27 min (30–52% B), 27–55 min (52–80% B), 55–65 min (80–100% B), and 65–72 min (100% B). The column temperature was 30 °C. The injection volume was 10 µL and the flow rate was 1 mL/min. MS data were recorded using an AB Sciex Triple TOF^TM^ 5600 system-MS/MS (AB SCIEX, Framingham, MA, USA) equipped with an electrospray ionization (ESI) source. The optimized MS conditions in positive-ion mode were set as follows: ion source temperature (TEM), 600 °C; flow rate of curtain gas (CUR), 40 psi; flow rate of nebulization gas (GS1) and flow rate of auxiliary gas (GS2), 60 psi; ion-spray voltage floating (ISVF), 5500 eV; collision energy (CE), 40 V. Data were acquired for each sample from 50 to 1500 Da. The data acquisition time was 72 min.

#### 4.4.3. Analysis of the Differential Constituents in AL from Different Habitats

The mass spectral identification of the main peaks in QC samples was inferred from the function of Formula Finder, XIC Manage by the Peakview software 1.2.0.3 (AB SCIEX) using reference substance mass spectral characteristics and literature reports of the same structure type, and the remaining substances were identified by MS-DIAL software 4.92 (URL: http://prime.psc.riken.jp/, accessed on 22 October 2022) in combination with its open-source database (URL: http://prime.psc.riken.jp/compms/msdial/main.html#MSP, accessed on 22 October 2022; scoring greater than 90).

MS-DIAL 4.92 software was also used to perform peak alignment and noise filtering for the raw mass spectrometry data. The results were further imported into the SIMCA-P 14.1 software (Umetrics AB, Umea, Sweden) with variates mean-centered by OPLS-DA. Lastly, variant metabolites were found using *t*-test (*p* < 0.05) and variable importance plot values (VIP > 1). The drawing was completed by the Origin 2019b software.

### 4.5. Extraction and Isolation

The liposoluble solution was extracted from the air-dried rhizomes (20 kg) with ethyl acetate by the percolation method at room temperature. The extract was concentrated under reduced pressure to yield a residue (1.5 kg). The extract (1.0 kg) was subjected to silica-gel column chromatography (Petroleum Ether-EtOAc-MeOH, 1:0-0:1-0:1, *v*/*v*/*v*). The fractions were pooled into seven subfractions according to the TLC analysis.

Among the Fr.1 flow group, the same flow was merged through TLC point plates to obtain three flow groups: Fr.1.1, Fr.1.2, and Fr.1.3. A large amount of nearly colorless crystals (brownish-red mother liquor) were produced in Fr.1.2 at room temperature, and the crystals were filtered to obtain about 1.03 g to yield compound **1**.

Within the Fr.2 flow group, four groups were obtained by merging the same flow through TLC point plates, namely Fr.2.1, Fr.2.2, Fr.2.3, and Fr.2.4. Among them, Fr.2.1 was successively washed with methanol, ethyl acetate, and dichloromethane to obtain a large amount of white crystals to produce about 313 mg of compound **2**. Fr.2.3 could precipitate and crystallize in different pre-tested solvents (n-hexane, petroleum ether, dichloromethane, ethyl acetate, anhydrous ethanol, methanol), and finally, anhydrous ethanol was added to the dry extract to precipitate colorless needle-like, oil-like crystals, which were identified as compound **3** (about 8.2 mg) by NMR.

Within the Fr.3 flow group, two flow groups, Fr.3.1 and Fr.3.2, were obtained by merging the same flow through TLC point plates. Among them, Fr.3.1 had white hairs after drying, and after adding n-hexane, feather-like crystal precipitates were formed. Under the thin-layer plate (sprayed with sulfuric acid and ethanol), red spots appeared, and this was identified as compound **4** (about 1.3 g) by NMR analysis.

Within the Fr.4 flow group, the same flow was merged through TLC point plates to obtain three flow groups: Fr.4.1, Fr.4.2, and Fr.4.3. Among them, Fr.4.1 underwent column chromatography (Si) again, merging the same flow components to obtain Fr.4.1.1, Fr.4.1.2, and Fr.4.1.3. After adding methanol, a large amount of white solids precipitated in Fr.4.1.2, and the white solids were taken out into the mixture of dichloromethane and methanol to precipitate nearly colorless snowflake crystals to yield compound **5** (about 1.1 g).

Fr.4.2 was subjected to column chromatography (Si) again, and the same flow fractions were merged to obtain Fr.4.2.1, Fr.4.2.2, and Fr.4.2.3. A white palisade-like oily solid precipitated from the bottom of the mother liquor stream (petroleum ether–ethyl acetate mixture) in Fr.4.2.2. There was no spot under an ordinary TLC and UV lamp, but a red spot was observed under the thin-layer plate (sulfuric acid–ethanol spray baking). This was identified as compound **6** (about 3.9 mg).

Fr.4.3 was subjected to column chromatography (Si) again, and the same flow components were merged to obtain Fr.4.3.1, Fr. 4.3.2, and Fr. 4.3.3. A colorless film of flaky solid crystals was separated from the stream in Fr.4.3.2. There were also no spots under ordinary TLC and UV light, and no spots under the thin-layer plate (sulfuric acid–ethanol spray baking). However, under the thin-layer plate (phosphomolybdic acid spray baking), there were blackish-green spots (Rf value was small under the developing agent of petroleum ether–ethyl acetate). This was identified as compound **7** (about 8.3 mg).

Within the Fr.6 flow group, the same flow was merged through TLC point plates to obtain three flow groups: Fr.6.1, Fr.6.2, and Fr.6.3. Methanol was added to remove insoluble matter in Fr.6.3, and petroleum ether was added after volatilization. The white suspension in the upper layer was obtained under ultrasound, and the suspension was taken out. After volatilization, needle-shaped crystals were separated from the bottom to yield compound **8** (about 133.8 mg).

## 5. Conclusions

In this study, eight compounds were separated and obtained as standard references; among these, the two diterpenes were first isolated and obtained from this genus, which updated the phytochemistry classification difference between the *Atractylodes* and *Atractylis* genera in Flora Reipublicae Popularis Sinicae.

LC-triple TOF-MS/MS and GC-MS were used to analyze the metabolites from ten different habitats of AL. In total, 106 overall chemical metabolites and 33 volatile metabolites were identified. The fragmentation pathways of sesquiterpenes and polyacetylenes were preliminarily deduced by the fragmentation behavior of the standard references.

OPLS-DA and *t*-tests were used to identify the different common chemical components of AL from different habitats. Finally, five overall differential metabolites and one volatile differential metabolite were selected out by screening. The contents of 3,5,11-tridecatriene-7,9-diyne-1,2-diacetate, (3Z,5E,11E)-tridecatriene-7,9-diynyl-1-O-(E)-ferulate, and atractylenolide I from the Maoshan area were higher than those from other habitats, while the contents of sucrose, 4(15),11-eudesmadiene, and β-eudesmol were lower.

In addition, the content of atractylone in the Maoshan area, which was the previous focus of geoherbalism research on AL, was indeed higher than that in other habitats. However, it was not a differential metabolite, indicating that other components may be more meaningful for distinguishing the geoherbalism of AL, which indirectly indicated the necessity of using metabolomics in this experiment.

In conclusion, these results can help us to better understand the componential differences of chemical metabolites in AL from different habitats and distinguish the geoherbalism chemical characteristics of AL as well as provide data for further exploring the geoherbalism molecular significance and environmental impacts of AL.

## Figures and Tables

**Table 1:** Identification of volatile metabolites in AL.

No.	RT	Compound Name	Formula	CAS	Types
**1**	6.36	β-patchoulene	C_15_H_24_	514-51-2	Sesquiterpenes
**2**	6.52	berkheyaradulene	C_15_H_24_	65372-78-3	Sesquiterpenes
**3**	7.10	γ-gurjunene	C_15_H_24_	22567-17-5	Sesquiterpenes
**4**	7.31	caryophyllene	C_15_H_24_	87-44-5	Sesquiterpenes
**5**	8.22	humulene	C_15_H_24_	6753-98-6	Sesquiterpenes
**6**	8.27	α-bulnesene	C_15_H_24_	3772-93-8	Sesquiterpenes
**7**	8.41	(+)-α-longipinene	C_15_H_24_	5989-08-2	Sesquiterpenes
**8**	8.54	trans-β-terpineol	C_10_H_18_O	7299-41-4	Monoterpenes
**9**	8.77	zingiberene	C_15_H_24_	495-60-3	Sesquiterpenes
**10**	8.83	α-selinene	C_15_H_24_	473-13-2	Sesquiterpenes
**11**	9.38	(E)-β-famesene	C_15_H_24_	18794-84-8	Sesquiterpenes
**12**	9.55	aromandendrene	C_15_H_24_	489-39-4	Sesquiterpenes
**13**	10.21	γ-elemene	C_15_H_24_	29873-99-2	Sesquiterpenes
**14**	11.02	dehydro-aromadendrene	C_15_H_22_	1000156-12-5	Sesquiterpenes
**15**	13.56	(−)-β-bourbonene	C_15_H_24_	5208-59-3	Sesquiterpenes
**16**	13.72	1,2,3,4,6,8α-hexahydro-1-isopropyl-4,7-dimethyl-naphthalene	C_15_H_24_	16728-99-7	Sesquiterpenes
**17**	14.06	elemol	C_15_H_26_O	639-99-6	Sesquiterpenes
**18**	14.98	atractylone	C_15_H_20_O	98309-94-5	Sesquiterpenes
**19**	15.88	γ-eudesmol	C_15_H_26_O	1209-71-8	Sesquiterpenes
**20**	16.10	agarospirol	C_15_H_26_O	1460-73-7	Sesquiterpenes
**21**	16.36	hinesol	C_15_H_26_O	23811-08-7	Sesquiterpenes
**22**	16.99	α-bisabolol	C_15_H_26_O	515-69-5	Sesquiterpenes
**23**	17.07	α-eudesmol	C_15_H_26_O	473-16-5	Sesquiterpenes
**24**	17.28	β-eudesmol	C_15_H_26_O	473-15-4	Sesquiterpenes
**25**	17.62	hedycaryol	C_15_H_26_O	21657-90-9	Sesquiterpenes
**26**	18.85	selina-4(15),7(11)-dien-8-one	C_15_H_22_O	54707-47-0	Sesquiterpenes
**27**	20.29	spathulenol	C_15_H_24_O	6750-60-3	Sesquiterpenes
**28**	22.11	7-ethynyl-1,4a-dimethyl-4a,5,6,7,8,8a-hexahydro-2(1H)-naphthalenone	C_14_H_18_O	55220-87-6	Others
**29**	26.22	atractylodin	C_13_H_10_O	55290-63-6	Polyethylenes
**30**	30.44	3β-acetoxy-atractylone	C_17_H_22_O_3_	61206-10-8	Sesquiterpene derivatives
**31**	31.09	prenortestosterone	C_18_H_26_O_2_	1089-78-7	Sesquiterpene derivatives
**32**	35.08	5-*O*-methyl-d-gluconic acid dimethylamide	C_9_H_19_NO_6_	13096-67-8	Others
**33**	36.67	atractylenolide I	C_15_H_18_O_2_	73069-13-3	Sesquiterpenes

**Table 2:** OPLS-DA results of AL samples from different habitats by GC-MS.

Samples	Model Verification Results			Permutation Results		Number of Characteristic Peaks with VIP > 1
	*R*^2^*X* (cum)	*R*^2^*Y* (cum)	*Q*^2^ (cum)	*R* ^2^	Q^2^	
S1, S2	0.999	0.983	0.952	0.236	−1.3	3
S1, S3	0.999	0.989	0.968	0.12	−1.34	3
S1, S4	0.998	0.991	0.974	0.145	−1.2	2
S1, S5	0.997	0.996	0.992	0.119	−1.44	3
S1, S6	0.997	0.984	0.952	0.281	−1.24	4
S1, S7	0.999	0.999	0.997	0.118	−1.33	2
S1, S8	0.998	0.993	0.98	0.138	−1.26	2
S1, S9	0.999	0.996	0.99	0.122	−1.26	2
S1, S10	0.999	0.999	0.998	0.146	−1.22	2

**Table 3:** Identification of 54 main metabolites in AL by LC-Triple TOF-MS/MS.

No.	Formula	Compound	RT	Adduct	MS	Error (ppm)	MS/MS	Ref.
**1**	C_15_H_22_	4(15),11-eudesmadiene-2H	61.79	+H	203.1789	−2.66	158.0630, 121.1010, 105.0720, 81.0680	[17]
**2**	C_15_H_24_	4(15),11-eudesmadiene	63.03	+H	205.1952	0.70	149.1328, 135.1178, 121.1027, 107.0874, 95.0881, 81.0737, 67.0590, 55.0605	[17]
**3** *	C_15_H_20_O	atractylone	67.59	+H	217.1585	−0.87	199.1491, 169.0938, 159.1141, 141.0691, 128.0635, 119.0844, 91.0585, 77.0407	[18]
**4** *	C_15_H_26_O	β-eudesmol	64.23	+H	223.2055	−0.72	207.0317, 191.0011, 175.9807, 162.9657, 133.0133, 111.0820, 93.0724, 55.0604	[19]
**5** *	C_15_H_26_O	hinesol	62.87	+H	223.2052	−1.97	207.0337, 191.0012, 162.9669, 107.0872, 73.0518, 69.0365	[20]
**6** *	C_15_H_18_O_2_	atractylenolide I	58.38	+H	231.1378	−0.61	213.1265, 185.1302, 155.0855, 141.0701, 128.0622, 115.0554, 105.0718, 91.0568, 79.0578	[18]
**7** *	C_15_H_20_O_2_	atractylenolides II	53.54	+H	233.1534	−0.86	215.1424, 187.1472, 145.1006, 131.0860, 115.0550, 105.0714, 91.0562, 79.0577	[18,19]
**8**	C_15_H_22_O	selina-4(15),7(11)-dien-8-one	61.47	+H	219.1737	−2.92	123.0811, 111.0822, 105.0717, 95.0877, 77.0423, 55.0606	[21]
**9**	C_15_H_22_O_2_	(5R,10S)-eudesm-4(15),7-diene-11-ol-9-one	57.01	+H	235.1696	1.45	179.1070, 57.0756	[22]
**10**	C_15_H_24_O_2_	eudesm-4(15),7-diene-9α,11-diol or isomer	65.01	+Na	259.1667	−0.58	175.0776, 161.0596, 137.0609	[22]
**11**	C_15_H_26_O_2_	eudesm-4(15)-ene-7α,11-diol or isomer	63.79	+H	239.2027	8.95	207.0301	[22]
**12** *	C_15_H_28_O_2_	cryptomeridiol	52.75	+K	279.1709	−6.10	261.1513, 201.0942, 185.0629, 149.0231, 96.9992, 71.9549, 56.9718	[23]
**13** *	C_15_H_20_O_3_	atractylenolides III	49.66	+H	249.1483	−0.92	231.1370, 213.1265, 175.0749, 163.0752, 161.0593, 105.0737, 91.0575	[19,24]
**14**	C_15_H_20_O_3_	atractylenolide III isomer	55.64	+H	249.1493	3.05	232.14615, 204.15298, 189.12842, 177.08931, 161.09698, 105.07215, 91.05746, 79.05825	[24]
**15**	C_15_H_24_O_3_	2-oxo-15-hydroxy-hinesol	62.95	+Na	275.1637	6.90	215.1436	[25]
**16**	C_15_H_26_O_3_	4α,7α-epoxyguaiane-10α,11-diol or isomer	59.25	+H	255.1950	−1.84	233.1012	[22]
**17**	C_15_H_28_O_3_	atractylol A	64.20	+K	295.1665	−1.69	226.9452, 207.0241, 205.1076, 177.0779, 161.0596, 158.9697, 133.0879, 121.0521	[25]
**18**	C_15_H_26_O_4_	10β,11β-epoxyguaiane-1α,4α,7α-triol	60.05	+K	309.1464	0.26	165.0685, 141.0686, 128.0595, 115.0574	[22]
**19**	C_18_H_24_O_5_	8-β-methoxy-atractylenolide IV	47.37	+H	321.1700	1.09	289.1408, 229.1240, 187.0723, 161.0605, 159.1154, 84.9646	[26]
**20**	C_20_H_30_O_7_	(5R,7R)-14-hydroxy-3,4-dehydrohinesolone-14-*O*-β-d xylopyranoside	60.35	+H	383.2060	−1.07	351.2198, 323.2198, 165.0596	[27]
**21**	C_21_H_36_O_7_	atractyloside C	31.23	+Na	423.2355	0.40	203.0535	[28]
**22**	C_21_H_38_O_8_	ophiopogonoside A	27.59	+Na	441.2458	−0.20	423.24942, 288.92529, 203.05148	[29]
**23**	C_21_H_36_O_9_	3,4,11,14-tetrahydroxyl-guaia-9-en-11-O-β-D-glucopyranoside	27.49	+Na	455.2254	0.55	275.1626, 203.0502	[30,31]
**24**	C_21_H_36_O_10_	atractyloside A	18.70	+Na	471.2199	−0.36	291.1540, 203.0513	[28]
**25**	C_21_H_38_O_10_	atractyloside B	17.90	+Na	473.2347	−2.16	293.1722, 203.0530	[28]
**26**	C_26_H_40_O_11_	(7R)-3,4-dehydrohinesolone-11-*O*-β-d-apiofuranosyl-(1→6)-β-d-glucopyranoside	31.42	+Na	551.2478	2.76	335.0966, 239.1451	[28]
**27**	C_27_H_46_O_12_	atractyloside D	26.99	+Na	585.2880	−0.26	405.2239, 203.0524	[28]
**28**	C_27_H_46_O_13_	(2R,3R,5R,7R,10S)-atractyloside G-2-*O*-β-d-glucopyranoside	29.70	+Na	601.2837	1.06	421.2214, 365.1131, 219.0903, 203.0547	[32]
**29**	C_29_H_46_O_14_	(5R,7R,10S)-6″-*O*-acetylatractyloside I or isomer	29.85	+H	619.2936	−3.89	575.1916, 439.2272	[33]
**30**	C_32_H_54_O_16_	atractyloside E	30.97	+Na	717.3315	1.52	537.2836, 509.0910	[28]
**31**	C_32_H_54_O_17_	atractyloside H	29.46	+Na	733.3226	−3.71	553.2485, 421.2140, 335.0881	[28]
**32**	C_37_H_62_O_20_	atractyloside F	65.27	+Na	849.3683	−5.12	471.1929, 455.1746, 387.1305, 267.0492, 213.0356	[28]
**33** *	C_13_H_10_O	atractylodin	63.55	+H	183.0801	−1.86	165.0698, 153.0683, 139.0518, 127.0500, 115.0558, 91.0597, 77.0375, 51.0293	[34]
**34**	C_13_H_10_O_2_	atractylodinol	58.95	+H	199.0760	3.21	165.0700, 154.0650, 127.0540	[34]
**35**	C_15_H_16_O_2_	(2Z,4E,10E)-dodeca-2,4,10-trien-6,8-diynyl acetate or isomer	61.18	+Na	251.1045	0.80	153.0688	[35]
**36**	C_15_H_12_O_3_	acetylatractylodinol	54.07	+Na	263.0678	−0.27	189.0186, 170.9241, 142.0810, 84.9651	[34]
**37**	C_17_H_18_O_4_	3,5,11-tridecatriene-7,9-diyne-1,2-diacetate	58.17	+Na	309.1103	1.84	249.0886, 227.1077, 167.0859, 165.0703, 152.0623, 141.0707, 129.0713, 128.0635, 115.0555, 91.0567	[36]
**38**	C_17_H_16_O_5_	1-(2-furyl)-(7E)-nonene-3,5-diyne-1,2-diacetate	54.03	+Na	323.0891	0.28	263.0691, 241.0867, 213.0908, 199.0760, 181.0651, 171.0803, 152.0622, 141.0704, 128.0635, 127.0551, 115.0557, 93.0371	[36]
**39**	C_18_H_20_O_4_	(4E,6E,12E)-Tetradecatriene-8,10-diyne-1,3-diyl diacetate	59.80	+Na	323.1253	−0.25	178.0777, 165.0700, 152.0628, 141.0707, 115.0556	[37]
**40**	C_17_H_18_O_5_	atractylodin+2 HAc	60.59	+Na	325.1040	−2.00	265.1201, 183.1150, 165.0700, 141.0691, 127.0530, 115.0566, 103.0599, 91.0562	[38]
**41**	C_18_H_20_O_5_	12,14-diacetate-2E,8E,10E-trien-4,6-diyne-1-ol	46.78	+Na	339.1206	0.88	197.0965, 178.0785, 153.0703, 141.0707, 128.0633, 115.0547, 91.0562, 77.0426	[35]
**42**	C_20_H_22_O_4_	3,5,11-tridecatriene-7,9-diyne-1,2-diacetate+-CH=CH-CH=	63.11	+Na	349.1419	2.61	303.11581, 289.12268, 225.15495, 209.0451, 165.06985, 127.05454	[38]
**43**	C_23_H_22_O_4_	(3Z,5E,11E)-tridecatriene-7,9-diynyl-1-*O*-(E)-ferulate	63.14	+Na	385.1409	−0.34	265.1475, 169.0960, 153.0646	[37]
**44**	C_16_H_24_O_7_	(8S)-decene-4,6-diyne-1,8-diol-8-*O*-β-d-glucopyranoside	53.19	+K	367.1145	−2.34	307.0940, 181.0577, 165.0711, 153.0696, 139.0541, 127.0542, 115.0534, 77.0478	[38]
**45**	C_19_H_24_O_8_	(8S,9R)-2E,10Z,12-tridecadiene-4,6-diyne-1,8,9-triol-8-*O*-β-d-glucopyranoside or isomer	30.28	+Na	403.1360	-0.84	203.0548, 201.0393	[38]
**46**	C_22_H_22_O_6_	Atractylodinol+-C≡C-CH_2_-+Rha	54.92	+H	383.1498	2.30	323.1218, 237.1132, 222.9705, 199.0979, 165.0704, 153.0717, 141.0691, 127.0530	[38]
**47**	C_17_H_22_O_8_S	atracthioenyneside B	49.06	+H	387.1117	2.27	341.1051, 183.0801, 181.0631, 153.0692, 141.0234, 127.0538, 115.0552	[38]
**48**	C_20_H_26_O_8_	(8E,10E)-tetradecatriene-4,6-diyne-3,12,14-triol-3-*O*-β-d-glucopyranoside or isomer	30.33	+K	433.1252	−1.62	365.1022, 201.0054	[38]
**49**	C_19_H_22_O_9_	(8R,9R)-8,9-dihydroxylatractylodinol-8-*O*-β-d-glucopyranoside or isomer	22.69	+H	395.1322	−3.69	233.0777, 185.0438	[38]
**50**	C_23_H_34_O_11_	(2E,10S)-tridecane-2-ene-4,6-diyne-1,10,13-triol-1,13-di-*O*-β-d-xylopyranoside	30.46	+H	487.2155	−3.88	335.0947, 275.0670	[38]
**51**	C_22_H_32_O_12_	(2E,8R)-decene-4,6-diyne-1,8-diol-*O*-di-β-d-glucopyranoside	28.32	+Na	511.1790	0.80	479.2397, 331.1006, 203.0734	[38]
**52**	C_24_H_32_O_12_	(8S,9R)-2E,10Z,12-tridecadiene-4,6-diyne-1,8,9-triol-8-*O*-β-d-apiofuranosyl-(1→6)-β-d-glucopyranoside or isomer	29.78	+Na	535.1783	−0.62	335.0952, 273.1044, 203.0588	[38]
**53**	C_25_H_34_O_12_	(2E,8E,10E,12R)-tetradeca-2,8,10-triene-4,6-diyne-1,2,14-triol-1-*O*-β-d-apiofuranosyl-(1→6)-β-d-glucopyranoside	27.41	+Na	549.1940	−0.46	387.1441, 189.0537	[38]
**54**	C_25_H_34_O_13_	(2E,8E,10E,12R)-tridecatriene-4,6-diyne-1,12,13-triol-1,12-di-*O*-β-d-glucopyranoside or isomer	28.25	+Na	565.1893	0.25	519.1195, 323.0466, 203.0549	[38]

Note: *: comparison with reference standards.

**Table 4:** Identification of other metabolites in AL by LC-Triple TOF-MS/MS.

No.	MW	Name	RT	Adduct	MS	Error (ppm)	MS/MS
**55**	C_16_H_28_O_7_	(1R,2R,4S)-2-hydroxy-1,8-cineole-β-d-glucopyranoside or isomer	28.88	+Na	355.1726	−0.28	203.0524
**56** *	C_20_H_32_O_2_	ent-kauran-16β-ol-19-al	61.69	+Na	327.2298	1.07	309.1835, 288.6881, 277.1044, 267.1307, 152.0590, 127.0091, 77.0088
**57**	C_30_H_48_O_3_	3-epiursolic acid	71.95	+Na	479.3487	−1.81	343.02658, 147.06119, 73.05546
**58**	C_41_H_66_O_13_	scabioside C	31.50	+Na	789.4460	8.16	788.4479
**59** *	C_32_H_52_O_2_	traxerol acetate	65.38	+Na	491.3873	2.75	445.2804, 431.3595, 269.1376, 236.1359, 193.1286, 161.1308
**60** *	C_29_H_50_O	β-sitosterol	69.04	+Na	437.3765	2.56	369.1972, 301.2097, 121.0989, 90.9812
**61**	C_35_H_58_O_6_	stigmasterol glucoside	29.45	+Na	597.4150	4.15	475.34491, 457.3175, 434.24863, 369.09949
**62**	C_15_H_16_O_3_	osthol	52.30	+Na	267.0998	2.36	165.0654, 115.0544
**63**	C_16_H_18_O_9_	isoscopoletin-6-*O*-β-d-glucopyranoside	24.47	+H	355.1031	2.08	163.0390, 145.0299, 135.0490, 117.0357, 89.0390
**64**	C_17_H_24_O_9_	syringin	53.08	+H	373.1508	3.99	357.0629, 168.9929, 252.9665, 220.9322, 178.9612, 90.9769, 73.0507
**65**	C_21_H_26_O_12_	umbelliferone-7-*O*-α-L-rhamnopyranosyl-(1→6)-β-d-glucopyranoside	21.76	+Na	493.1319	0.51	331.1022
**66**	C_22_H_28_O_13_	scopoletin-7-*O*-α-L-rhamnopyranosyl-(1→6)-β-d-glucopyranoside	30.95	+K	539.1165	0.65	521.1151, 377.0853, 359.0731, 331.0757, 163.0388,185.0177, 197.0404, 163.0388
**67**	C_10_H_8_O_4_	scopoletin	29.57	+H	193.0499	1.86	178.02585, 161.01437, 150.03203, 133.03062, 122.03758, 105.03378, 94.04397, 77.03789
**68**	C_11_H_10_O_4_	6,7-dimethoxychromen-2-one	31.72	+H	207.0654	1.01	191.03391, 179.07031, 163.03935, 151.07645, 145.02959, 135.04448, 118.04246, 107.05118, 91.05722, 89.04136, 77.04197
**69**	C_25_H_32_O_11_	3′,9,9′-trihydroxy-3-methoxyI-7,8-dihydrobenzofuran-1′-propanolneolignan-4-*O*-β-d-glucopyranoside	27.78	+Na	531.1831	−1.09	513.1563, 501.1827, 339.1278, 305.0925, 185.0434
**70**	C_26_H_34_O_11_	(+)-Isolariciresinol-9′-*O*-β-d-glucopyranoside	29.43	+Na	545.1995	0.31	383.1396
**71**	C_26_H_36_O_11_	secoisolariciresinol-4-*O*-β-d-glucopyranoside or isomer	29.08	+Na	547.2143	−1.24	385.1695185.0332, 159.0377
**72**	C_26_H_36_O_12_	(7R,8S)-5-methoxyldihydrodehydrodiconiferylalconol-4-*O*-β-d-glucopyranoside	28.76	+Na	563.2093	−1.07	353.1351, 204.0735
**73**	C_27_H_36_O_13_	longifloroside B	28.40	+Na	591.2042	−1.03	429.1518, 351.1049, 232.0716
**74**	C_27_H_38_O_13_	longifloroside B+2H	28.74	+Na	593.2188	−2.78	431.1657, 234.0896
**75**	C_17_H_22_O_10_	(7E)-sinapate-4-*O*-β-d-glucopyranoside	44.90	+K	425.0841	−0.85	193.0448, 149.0239
**76**	C_12_H_14_O_4_	diethyl phthalate	59.72	+H	223.0966	0.49	207.0329, 191.0018, 149.0247, 73.0510
**77**	C_18_H_26_O_2_	2-[(2′E)-3′,7′-dimethyl-2′, 6′-octadienyl]-4-methoxy-6-methyl phenol	63.60	+H	275.2003	−0.94	205.1229, 191.1086, 177.0946, 151.0776, 123.0760
**78**	C_19_H_28_O_10_	phenylmethanol 7-*O*-α-l-rhamnopyranosyl-(1→6)-β-d-glucopyranoside	27.38	+H	417.1735	−4.84	347.0970, 271.1149
**79**	C_17_H_22_O_5_	trans-2-hydroxyisoxypropyl-3-hydroxy-7-isopentene-2,3-dihydrobenzofuran-5-carboxylic acid	44.75	+Na	329.1359	−0.30	289.1481, 271.1285, 239.0882, 205.0832, 179.0333, 163.0374, 149.0283, 119.0541, 69.0739
**80**	C_24_H_38_O_4_	bis(2-ethylhexyl) phthalate	69.00	+H	391.2841	−0.49	279.1599, 149.0244, 121.03027, 71.09079
**81**	C_5_H_11_NO_2_	valine	2.54	+H	118.0866	2.88	58.0709
**82**	C_5_H_7_NO_3_	5-oxo-l-proline	2.40	+H	130.0501	1.77	84.0478, 56.0558
**83**	C_4_H_8_N_2_O_3_	asparagine	2.37	+H	133.0611	2.48	116.04055, 87.05751, 74.02784, 53.01179
**84**	C_5_H_11_N_3_O_2_	4-guanidino-butanoate	2.24	+H	146.0924	0.00	128.08316, 104.0715, 86.06216, 69.03831, 60.06041
**85**	C_6_H_9_N_3_O_2_	histidine	1.84	+H	156.0767	−0.32	110.07291, 93.04785, 83.06383, 66.04191, 56.05435
**86**	C_7_H_15_NO_3_	carnitine	1.97	+H	162.1123	−1.05	103.04109, 85.03056, 58.07046
**87**	C_6_H_14_N_4_O_2_	arginine	1.83	+H	175.1191	0.57	70.0694, 60.0610
**88**	C_7_H_16_N_4_O_2_	targinine	1.89	+H	189.1345	−0.53	144.112, 116.07196, 70.06947, 57.05048
**89**	C_8_H_18_N_4_O_2_	symmetric dimethylarginine	1.93	+H	203.1502	−0.25	172.10738, 158.12762, 133.10193, 116.07109, 88.08673, 70.06877
**90**	C_12_H_22_O_11_	sucrose	2.77	+Na	365.1055	0.19	347.0935, 275.0727, 203.0522, 185.0416
**91**	C_18_H_32_O_16_	melezitose	2.89	+Na	527.1581	−0.30	365.1039, 347.0937, 275.0712, 203.0515, 185.0409
**92**	C_24_H_42_O_21_	stachyose	2.96	+Na	689.2097	−1.97	527.1546, 509.1450, 365.1038, 347.0933, 203.0519, 185.0417
**93**	C_10_H_13_N_5_O_4_	adenosine	3.18	+H	268.1044	1.38	136.0619, 119.034
**94**	C_5_H_5_N_5_	adenine	2.51	+H	136.0623	3.90	119.03603, 92.02648, 77.01751, 65.01727, 55.03499
**95**	C_5_H_7_N_3_O	5-methylcytosine	2.72	+H	126.0667	4.05	109.0421, 83.0635, 54.0397
**96**	C_4_H_5_N_3_O	cytosine	2.23	+H	112.0508	2.32	95.02696, 67.03505, 52.02482
**97**	C_6_H_6_N_2_O	nicotinamide	4.07	+H	123.0558	4.14	106.03112, 80.05253, 53.04439
**98**	C_22_H_43_NO	erucamide	70.06	+H	338.3414	−1.00	321.3154, 303.30508, 163.15129, 149.13307, 135.1179, 97.10375, 83.08909
**99**	C_14_H_31_NO	*N*,*N*-dimethyldodecylamine *N*-oxide	39.43	+H	230.2481	1.13	212.2370, 58.0706
**100**	C_18_H_24_O_2_	galaxolidone	66.72	+H	273.1847	−0.77	255.1843
**101**	C_16_H_35_NO_2_	lauryldiethanolamine	36.77	+H	274.2744	1.24	256.26328, 230.24826, 106.08877, 88.07969, 70.07063, 57.07623
**102**	C_18_H_39_NO_2_	2,2′-(tetradecylimino) diethanol	43.03	+H	302.3053	−0.20	284.29459, 258.27921, 106.08905, 88.07846
**103**	C_19_H_20_O_5_	6a,7,8-trihydroxy-3-methyl-3,4,5,6,7,12a-hexahydro-2H-benzo[a]anthracene-1,12-dione	2.98	+Na	351.1192	−3.13	333.0990
**104**	C_19_H_20_O_6_	lasepitin	2.67	Na	367.1137	−5.6	269.9946, 205.0700, 88.0824
**105**	C_27_H_50_O_6_	tricaprylin	69.85	+Na	493.3487	−2.55	349.2350, 327.2496, 127.1130
**106**	C_32_H_54_O_15_	1,3,4,6-tetrakis-*O*-(3-methylbutanoyl)-β-d-fructofuranosyl-α-d-glucopyranoside	62.14	+Na	701.3349	−1.6	539.2818

Note: *: comparison with reference standards.

**Table 5:** OPLS-DA results of AL samples from different habitats by LC-Triple TOF-MS/MS.

Samples	Model Verification Results			Permutation Results		Number of Characteristic Peaks with VIP > 1
	*R*^2^*X* (cum)	*R*^2^*Y* (cum)	*Q*^2^ (cum)	*R* ^2^	*Q* ^2^	
S1, S2	0.978	1	1	0.719	−0.518	12
S1, S3	0.982	0.999	0.998	0.142	−1.02	15
S1, S4	0.955	1	0.998	0.856	−0.395	13
S1, S5	0.962	0.999	0.998	0.664	−0.705	17
S1, S6	0.953	0.999	0.996	0.356	−0.816	13
S1, S7	0.97	0.999	0.996	0.64	−0.661	16
S1, S8	0.934	1	0.996	0.777	−0.272	13
S1, S9	0.974	0.999	0.998	0.555	−0.774	15
S1, S10	0.87	0.999	0.987	0.359	−0.35	17

**Table 6:** Information on AL samples from different varieties and habitats.

No.	Habitat	Longitude and Latitude (°)
S1	Maoshan Region, Jiangsu	119.315350, 31.781150
S2	Huoshan, Luan, Anhui	116.33269, 31.39279
S3	Xixia, Henan	111.47244, 33.3056
S4	Tongbai, Henan	113.42892, 32.37913
S5	Guangshan, Xinyang, Henan	114.91878, 32.00997
S6	Yingshan, Huanggang, Hubei	115.68143, 30.73518
S7	Suizhou, Hubei	113.3712, 31.71615
S8	Luotian, Huanggang, Hubei	115.39927, 30.78399
S9	Yunxi, Shiyan, Hubei	110.42588, 32.99306
S10	Yecun, Shangluo, Shanxi	110.1353, 33.7643

## Data Availability

The data presented in this study are available within the article.

## Sample Availability

Samples of AL are available from the authors.
